# The Association between HIV Infection, Antiretroviral Therapy and Cervical Squamous Intraepithelial Lesions in South Western Nigerian Women

**DOI:** 10.1371/journal.pone.0097150

**Published:** 2014-05-08

**Authors:** Oliver Chukwujekwu Ezechi, Karen Odberg Pettersson, Clement Abu Okolo, Innocent Achaya O. Ujah, Per Olof Ostergren

**Affiliations:** 1 Clinical Sciences Division, Nigerian Institute of Medical Research Yaba, Lagos, Nigeria; 2 Social Medicine and Global Health, Faculty of Medicine, Lund University, Lund Sweden; 3 Department of Pathology, College of Medicine, University of Ibadan, Ibadan, Nigeria; Centro de Biología Molecular Severo Ochoa (CSIC-UAM), Spain

## Abstract

**Introduction:**

Findings from studies that evaluated the effect of antiretroviral drug use on the development of cervical squamous intraepithelial lesion differed in their conclusions. This study investigated the association between HIV infection, antiretroviral drug use and cervical squamous intraepithelial lesion in a high HIV and cervical cancer burden setting- Nigeria.

**Methods:**

A cross sectional study among 1140 women of known HIV status enrolled in a randomised study to determine the test characteristics of visual inspection in detecting cytology diagnosed squamous intraepithelial lesion. Multivariate analysis was used to determine the association between HIV infection, antiretroviral drug use and the twin outcome variables of cervical squamous intraepithelial lesion (SIL) and High grade squamous intraepithelial lesion (HSIL) while controlling for confounders.

**Results:**

Prevalence of cervical squamous intraepithelial lesion was 8.5%, with a higher prevalence of 14.3% in HIV positive compared to 3.3% in HIV negative women (aOR: 5.4; 95% CI: 2.9–8.8). Not using antiretroviral drugs was found to be associated with an increased risk of SIL (aOR: 2.1; 95% CI: 1.4–3.5) and HSIL (aOR: 2.6; 95% CI: 1.1–6.4). Participants who had a CD4 cell count <200 cells/mm^3^, were also found to be at increased risk for SIL (aOR: 1.9; 95% CI: 1.1–5.9) and HSIL (aOR: 5.7; 95% CI: 1.1–7.2).

**Conclusion:**

HIV infection and severe immunosuppression were found to be associated with increased risk of cervical squamous intraepithelial lesion but not viral load. For the first time, in the West African sub-region with specific HIV type and strains, we established the protective effect of antiretroviral drug use against the development of SIL. Integration of cervical cancer screening programme into HIV services and early initiation of antiretroviral drug in HIV positive women especially those with severe immune-suppression could therefore prove to be useful in preventing and controlling cervical cancer development in HIV positive women.

## Introduction

The introduction of, and improved access to highly active antiretroviral therapy (HAART) has had a dramatic impact on morbidity and mortality among individuals living with human immunodeficiency virus [1], [2], [3]. However, information on the impact of HAART on the natural history and treatment outcomes of HIV-associated malignancies are limited[4]. The possibility of direct and indirect roles of HIV in HIV-related carcinogenesis suggests that antiretroviral therapy may be an important component of the treatment strategy for some AIDS defining malignancies [4], [5], [6].

Despite availability of studies that examined the effects of antiretroviral drug use on the course of cervical lesions in HIV positive women, the impact of the use of antiretroviral drug on the course of cervical lesions remains somewhat unclear [7]–[13]. While some studies [10]–[12], [15], [16]. reported an association between antiretroviral drugs use and a lower incidence of cervical lesions, others have reported no beneficial effect of antiretroviral drug use on the incidence of cervical lesions [7]–[12], [17], [18]. The inconsistency of findings in the cited studies might be the effect of small sample size, differing analytic techniques, viral diversity as well as timing and duration of treatment with antiretroviral drugs [14], [17].

It is now more than 10 years since the Government of Nigeria introduced an antiretroviral drugs access programme. Over half a million adults have initiated treatment, 60% of whom are women[1]. Unfortunately, little or no information exists on the impact of antiretroviral drug use on cervical lesions among women benefitting from the programme. The focus of published studies has been on prevalence and burden of cervical precancerous and invasive lesions among HIV-infected Nigerian women [19]–[22]. Detailed literature search identified no study in our sub region that has evaluated the effect of HIV infection, immunosuppression and antiretroviral drug use on the burden and distribution of cervical squamous intraepithelial lesions.

In this study, we investigated the association between HIV infection, immunosuppression, antiretroviral drug use and cervical squamous intraepithelial lesions in a setting endemic for both HIV and cervical cancer.

## Methods

### Ethical Considerations

Approval for the study was obtained from the Institutional Review Board, Nigerian Institute of Medical Research (NIMR), Lagos Nigeria. A written informed consent was obtained from the women invited to be part of the study after detailed information about the study. Impartial witnesses who were not members of study staff assisted the consenting process for low literates.

### Study Design and Setting

A cross-sectional study among 1140 women of known HIV status enrolled in a study to evaluate the test characteristics of direct visual inspection in detecting cytology diagnosed cervical squamous intraepithelial lesion in Nigerian women of known HIV status.

Participants were recruited at the cervical cancer screening clinic, NIMR, Lagos, and from10 communities of Lagos and Ogun States of Nigeria during community cervical cancer screening outreach programmes. Women who presented for cervical cancer screening at the clinic and during the community outreach programmes were screened for eligibility for recruitment into the main study.

NIMR, the apex medical research institution in Nigeria, hosts a HIV treatment Centre that currently provides comprehensive HIV services for over 20,000 patients of whom 62.9% are women. Approximately 80% of the Centre’s patients come from Lagos and Ogun state and all services are provided free of charge.

### Study Population

The study population consists of adult females aged 18 years and above, seen either at the cervical cancer screening clinic, NIMR, Lagos or during the community cervical cancer screening outreach programmes.

### Cytology Sample Collection and Analysis

Midwives and physicians trained specifically for this study performed all clinical examinations and the sample collection for Cervical Pap tests. Cervical samples collected on Ayres spatula and smeared on the slides were immediately fixed with commercial fixator before transportation to Department of Anatomic Pathology, University College Hospital Ibadan, Nigeria for interpretation according to Bethesda system. The cytopathologists who performed the cytological analyses were blinded to the participant’s HIV status. A senior pathologist read all tests originally classified as abnormal and 15% of those classified as normal. All slides were pre-coded with the participants’ study number before samples were taken. In the event of disagreement between the cytopathologist’s and the senior pathologist’s report, the slides were sent to another senior pathologist for an independent review. For all such cases, that review constituted the final diagnosis.

### Laboratory Tests

HIV test was conducted according to Nigerian National HIV testing and counselling guidelines in all women before enrolment into the study. Diagnosis was based on positive test on double ELISA based algorithm.

Viral load and CD4 cell count tests were conducted at the Human Virology Laboratory, NIMR, Lagos. Whole blood of the HIV positive women was used to perform CD4 assay, using the Cyflow Counter and Kits (Partec, Germany) according to the manufacturer’s instructions. The viral load assay was performed using Roche Amplicor HIV-1 monitor test (version 1.5) according to manufacturer’s instruction.

### Data Collection and Analysis

Information on the socio-demographic, sexual and reproductive characteristics, HIV treatment history was collected by trained midwives and physicians using a study record form. The laboratory information (HIV status, CD4 cell count, HIV plasma viral load and cytology) were extracted from the participant’s laboratory results and entered into the relevant portion of the study record form by trained staff. The entered information was cross-checked with the laboratory results to avoid entry error.

Information entered into the study record forms were doubly entered into study database by two trained data clerks. Information from the two databases was compared and any observed disparities were corrected using the individual case record forms. Coded information were then analysed using the SPSS version 19.0 (SPSS Inc. Chicago, IL) statistical package. The main outcome variables were cervical squamous intraepithelial lesions (SIL) and High grade squamous intraepithelial lesions (HSIL). For the analysis and as per Bethesda classification, atypical squamous cell of undetermined significance was distinguished from either squamous intraepithelial lesion or high grade squamous intraepithelial lesion and treated as a distinct category. First level analysis was conducted to determine the overall prevalence of cervical cell abnormality, SIL and HSIL, among the women and the prevalence of SIL and HSIL by HIV status. Second level analysis was then performed to assess the association between socio-demographic factors, sexual and reproductive characteristics, HIV treatment history and the two outcome variables. All factors independently associated with SIL and HSIL at the second level analysis were considered confounding variables and introduced in a step-wise manner in three different models of multivariate regression. In the first step, we started with the crude association between the cofounding variables and outcome variables adjusted by age and marital status (Model 1). Secondly, we adjusted for HIV status and treatment (Model 2) and finally we adjusted for other variables that were shown to be significantly associated with SIL and HSIL (Model 3). The results are presented as Odds ratios (OR) or adjusted ORs and their 95% confidence intervals (CI).

### Determination of Sample Size

The prevalence of abnormal cervical cytology of 57.5% among women reported by Agaba et al in Jos, Nigeria was used as a reference value for the calculation of sample size [19]. Though this prevalence is considerably higher than a range of 5.0–6.8% [23]–[25] reported among HIV negative women in Nigeria, the higher prevalence was used because of the large proportion of HIV positives in this study. The minimum sample size for a statistically meaningful deduction was determined using the formula: N = Zα^2^P (1-P)/d2, where Zα is the Z statistic for a 95% confidence level, N is the sample size, p is the prevalence of cervical cell abnormality, and d is the precision. Based on this calculation, screening 720 women aged 18 years and above was considered sufficient for identifying a reliable estimate of the prevalence of women with cervical cell abnormality (CCA).

### Definition of Variables

- HIV treatment status: The use of antiretroviral therapy among HIV positive women for at least 3 months.
- Viral load: The level of HIV-1 RNA copies/mL of plasma measured by Roche Ampliclor HIV-1 monitor test (version 1.5). HIV viral load value during analyses was dichotomized into two using 1000 copies regarded as the threshold for low viraemia.
- CD4 Cell count: The level of the body’s immunity measured as the number of CD4 T lymphocytes per mm^3^ of blood by Cyflow Counter and Kits. CD4 cell count during analyses was dichotomized into two using 200 cell/mm^3^ as cut off point. The immune system is adjudged as severely compromised at levels below 200.
- Cervical cell abnormality (CCA): Any of atypical squamous cell, low grade squamous intraepithelial lesion, high grade squamous intraepithelial lesion or invasive cancer found in the cytology.
- Atypical squamous cell abnormality of undetermined significance (ASC-US)[26]: Cellular abnormalities that are more marked than those attributable to reactive changes but that quantitatively or qualitatively fall short of a definitive diagnosis of low-grade squamous intraepithelial lesion. Not included as squamous intraepithelial lesion and distinguished from Atypical Squamous Cell cannot exclude high grade squamous intraepithelial lesion (ASC-HSIL).
- Low grade Squamous Intraepithelial Lesion (LSIL))[26]: Encompassing: HPV/mild dysplasia/cervical intraepithelial neoplasia (CIN) 1.
- High grade Squamous Intraepithelial Lesion (HSIL))[26]: Encompassing: moderate and severe dysplasia, carcinoma in situ (CIS)/CIN 2 and CIN 3, with features suspicious for invasion.
- Squamous Intraepithelial Lesion (SIL) )[26]: Encompasses all cases of LSIL and HSIL.
- Age at first intercourse: The age of the participants at her first penetrative vaginal intercourse.
- Life time sexual partners: The total number of participants’ sexual partner at the time of recruitment.
- Duration of ARV drug use: The time elapsed since commencement of antiretroviral therapy.

## Results

A total of 1156 women were enrolled into the study. The 1140 (98.6%; 531 HIV positives and 609 HIV negatives) women with cytology reports were included in the analysis. Cytology results for the remaining 23 women were excluded due to failure of collecting smear because of vaginal atresia/uncooperative participant (5) and participant’s refusal to allow pelvic examination and specimen collection after consenting process (8).

### Cytology Quality Control

A total of 358 (31.4%) slides were reviewed as per protocol. There were discordant results in only seventeen (4.7%) of cases originally diagnosed as ASC-US. Independent review concurred in ten (58.8%), downgraded as normal in six (35.3%) and upgraded one in (5.9%) to low grade squamous intraepithelial lesions. The original review accuracy was thus 98.5%.

### Participants’ Characteristics

The demographic and biologic characteristics of the 1140 participants with cytology reports are shown in table 1. About two-third of the women were older than 35 years of age (60.9%) with a median age of 37 years [IQR: 31–45]. A majority of the women were married (66.9%), residing in urban community (57.6%) and had completed at least a secondary education (77.0%). Most women were employed (85.1%) and had at least one child (81.5%). Sexual debut occurred between the ages 15 and 24 years for most women (81.2%, mean: 20.4±3.9). Over 70% of the women reported total lifetime sexual partners of at least 2 (77.1%, mean: 3.0±2.5). Among the HIV positive participants, the majority had CD4 cell count above 200 cells/mm^3^ (91.9%, mean: 532±268,4) and viral load less than 1000 copies per ml (73.1%, mean: 94676.8±692581.9). Most of the HIV positive women in the study were on antiretroviral drugs (76.1%). Of the 404 HIV positive women on antiretroviral drugs, 231(57.2%) have been on treatment for more than 3 years. Further analysis on the relationship between age at sexual debut and number of life time sexual partners showed that 52.4% (332) of the 633 women that had sexual debut after 20 years reported having had at least four sexual partners compared to only 28.4% (114/401) of 401 that had sexual debut before 20 years (p = 0.00; OR: 2.8; 95% CI: 2.1–3.7).

**Table 1:** Socio-demographic and biologic characteristics of the 1133 women in the study.

Characteristics	Number of women [n = 1140(%)]
**Age (years; 1135)**	
18–24	48 (4.2)
25–34	395 (34.8)
35–44	358 (31.5)
≥45	334(29.4)
**Educational Status (1140)**	
None	89(7.8)
Primary	173 (15.2)
Secondary	446(39.1)
Tertiary	432(37.9)
**Marital status (1134)**	
Never married	192 (16.9)
Married	759 (66.9)
Divorced/Separated	60(5.3)
Widow	123(10.8)
**Ethnic Group (1126**)	
Yoruba	378(33.6)
Igbo	301(26.7)
Hausa/Fulani	96(8.5)
Northern Minority	113(10.0)
Southern Minority	226(20.1)
others	9(0.8)
**Residence(1140)**	
Urban	657 (57.6)
Rural	483 (42.4)
**Occupation(1136)**	
Unemployed	163 (14.3)
Unskilled	380(33.5)
Skilled	449(39.5)
Professionals/Business Executive	144(12.7)
**Age at first intercourse (years;1034)**	
<15	26(2.5)
15–19	375(36.3)
20–24	465(44.9)
≥25	168 (16.2)
**Life time sexual partners (1063)**	
1	313 (22.9)
2–4	571 (53.7)
5–9	139(13.1)
≥10	40(3.7)
**Parity (1108)**	
0	205 (18.5)
1–4	731 (66.0)
≥5	172 (15.5)
**HIV status (1140)**	
Negative	609(53.4)
Positive	531(46.6)
**CD4 cell count (Cells/mm^3^)** *	
<200	43 (8.1)
200–499	224 (42.2)
>500	264 (49.7)
**HIV viral load (Copies/ml)** *	
<1000	387 (73.1)
1000–9999	35 (6.4)
>10,000	109 (20.5
**Antiretroviral drug use** *	
Not on drugs	127 (23.9)
On drugs	404 (76.1)
**Duration of ARV drug use** *	
<3 years	228(42.9)
3–5 years	161(30.3)
Above 5 years	142(26.7)

*HIV positive women only.

### Prevalence and Distribution of Cervical Squamous Intraepithelial Lesions

Two hundred and twenty (19.3%) women out of the 1140 women in the study had a cytology report of CCA. Of the 220 women with CCA cytology report, 121 (55.0%) were reported as atypical squamous cell abnormality of undetermined significance (ASC-US. Ninety-six (44.0%) were reported as squamous intraepithelial lesion (SIL) and two (0.9%) as invasive cancer and atypical glandular cell abnormality in one (0.5%) woman. Of the 96 women with SIL cytology report, 67 (69.8%) were further classified as low grade squamous intraepithelial lesion and 29 (30.2%) as high grade squamous intraepithelial lesion (HSIL). The prevalence of SIL and HSIL among the 1140 women in the study was thus 8.4% (67/1140) and 2.5% (29/1140) respectively. While 76 (14.3%) and 23 (4.3%) of the 531 HIV positive women were reported to have an abnormal cytology finding of SIL and HSIL respectively, 20 (3.3%) and six (1.0%) of the 609 HIV negative women were found to have SIL and HSIL respectively. The three women with invasive cancer (2) and atypical glandular cell abnormality (1) were all HIV-positive.

### Distribution of Sociodemographic and Biological Characteristics by Cervical Smear Status

Table 2 shows the prevalence of SIL and HSIL by exposure variable. Participants who reported more than one life time sexual partner (OR: 2.9; 95% CI: 1.6–5.4), were unmarried (OR: 1.6; 95% CI: 1.0–2.6) and initiated sex after age of 20 years (OR: 17.7; 95% CI: 6.2–57.0) were more likely to have cervical smear report of SIL. HIV positive status (OR: 5.8; 95% CI: 3.3–10.0), non-use of antiretroviral drugs by HIV positives (OR: 2.0; 95% CI: 1.3–3.7), viral load more than 1000 copies/ml (OR: 2.0; 95% CI: 1.2–3.4), and CD4 cells count of less than 200 cells/mm^3^ (OR: 3.8; 95% CI: 1.9–7.9) were also found to be at increased risk of SIL.

**Table 2:** The relationship between sociodemographic and biological variables and cervical smear status.

Characteristics	Normal smear[n = 913(%)]	SIL [n = 96(%)]	HSIL[n = 29(%)]	SIL[Odd Ratio(95% CI)]	HSIL[Odd Ration (95% CI)]
**Age**					
Less than 35	347(38.1)	43 (44.8)	14(48.3)	1.31(0.8–2.1)	1.5(0.7–3.4)
≥35	562(61.9)	53(55.2)	15(51.7)	1(ref)	1(ref)
**Educational Status**					
<Secondary	200(21.9)	28(28.7)	8(27.6)	1.4(0.9–2.4)	1.4(0.5–3.3)
≥Secondary	713(78.1)	68(71.3)	21(72.4)	1(ref)	1(ref)
**Marital status**					
Unmarried	285(31.3)	41(42.7)	12(41.4)	1.6(1.0–2.6)	1.6(0.7–3.5)
Married	625(68.7)	55(57.3)	17(58.6)	1(ref)	1(ref)
**Age at first intercourse**					
<20	397(2.7)	4(4.2)	2(6.9)	1(ref)	1(ref)
≥20	516(18.5)	92(95.8)	27(93.1)	17.7(6.2–57.0)	10.4(2.4–63.5)
**Life time sexual partners**					
1	313(35.2)	15(15.6)	4(13.8)	1(ref)	1(ref)
≥2	575(52.7)	81(84.4)	25(86.2)	2.9(1.6–5.4)	3.4(1.1–11.6
**HIV status**					
Negative	551(60.4)	20(20.8)	5(20.0)	1(ref)	1(ref)
Positive	362(39.6)	76(79.2)	20(80.0)	5.8(3.3–10.0)	6.1(2.1–18.7)
**CD4 cell Count** *					
<200	22(6.1)	16(16.7)	5(20.0)	3.8(1.9–7.9)	4.0(1.2–12.6)
≥200	413 (93.3)	80(83.3)	24(80.0)	1(ref)	1(ref)
**HIV viral load** *					
<1000	282(77.9)	61(63.5)	14(56.0)	1(ref)	1(ref)
≥1000	80(22.1)	35(36.5)	11(44.0)	2.0(1.2–3.4)	2.8(1.1–6.8)
**ARV drug use** *					
No	74(20.1)	32(33.3)	10(40.0)	2.0(1.3–3.7)	2.7(1.1–6.6)
Yes	295(79.9)	64(66.7)	15(60.0)	1(ref)	1(ref)

*HIV positive women only; SIL: Squamous Intraepithelial lesion; HSIL: High grade Squamous Intraepithelial lesion.

Women living with HIV infection (OR: 6.1; 95% CI: 2.1–18.7), were not on antiretroviral drugs (OR: 2.7; 95% CI: 1.1–6.6) had a CD4 count less than 200 cells/mm^3^ (OR: 4.0; 95% CI: 12.6) and HIV viral load more than 1000 copies/ml (OR: 2.8; 95% CI: 1.1–6.8) were found to be at increased risk of HSIL, similar to the pattern observed for SIL. Women who reported more than one life time sexual partner (OR: 3.4; 95% CI: 1.1–11.6) or initiated sex at age of 20 years and above (OR: 8.9; 95% CI: 2.0–54.3) were also found to be at increased risk of HSIL. However when the effect of the number of life time sexual partnership, was controlled for in a multivariate logistic regression, the association between age at sexual debut above 20 years and both SIL (OR: 9.3; 95% CI: 0.9–19.7) and HSIL (OR: 3.9; 95% CI: 0.5–27.9) was lost.

### Association between HIV Infection, Immunodeficiency, Antiretroviral Therapy and Cervical Squamous Intraepithelial Lesion

The association between HIV infection, immunodeficiency, use of antiretroviral therapy and cervical squamous intraepithelial lesion was determined by comparing the distribution of these effect variables among the women with SIL/HSIL and those without SIL/HSIL. The results (crude odd ratios) determined at bivariate analysis (table 2) were further refined at multivariate analysis. In order to assess the effect of different types of exposures on the outcomes, we analyzed and reported our analysis in three models (table 3). The increased risks for SIL (cOR: 5.8; 95% CI: 3.3–10.0) and HSIL (cOR: 6.1; 95% CI: 2.1–18.71), respectively, observed among HIV positive women relative to HIV negative women in the bivariate analysis were confirmed for both SIL (aOR: 5.4; 95% CI: 2.9–8.8) and HSIL (aOR: 5.7; 95% CI: 2.4–10.1) after adjustment were made for confounders in the three models.

**Table 3:** Relationships between HIV and HIV related variables and squamous intraepithelial lesion (SIL) and high grade squamous intraepithelial lesion (HSIL) after controlling for confounders in a multivariate logistic regression models.

Exposure variables and confounders	Crude Ratio [OR (95% CI)]		Model 1 [OR (95% CI)]		Model 2 [OR (95% CI)]		Model 3 [OR(95%CI)]	
	SIL	HSIL	SIL	HSIL	SIL	HSIL	SIL	HSIL
**HIV Positive status**	5.8(3.3–10.0)	6.1(2.1–18.7)	5.6(3.2–9.3)	6.0 (2.0–16.3)	5.4(3.2–9.1)	5.8 (2.6–12.3)	5.5(3.3–9.9)	5.6(3.1–11.7)
Age^a^ and marital status^b^	**-**	**-**	5.2(3.1–9.1)	5.6 (2.6–11.3)	5.1(3.0–8.9)	5.1 (2.2–10.5)	5.1(3.4–8.1)	5.1(2.2–10.5)
Age at first intercourse^c^	**-**	**-**	**-**	**-**	4.7(1.9–9.5)	5.7 (2.1–9.1)	4.8(2.1–9.3)	5.7(2.1–9.9)
Life time sexual partner^d^	**-**	**-**	**-**	**-**	**-**	**-**	5.4(2.9–8.8)	5.7(2.4–10.1)
**Use of ARV drugs** *	2.0(1.3–3.7)	2.7(1.1–6.6)	1.9(1.0–3.3)	2.7(1.2–6.8)	2.0(1.0–3.7)	2.5(0.8–6.7)	2.0(1.2–3.5)	2.6(1.0–6.7)
Age and marital status	**-**	**-**	2.0(1.2–3.5)	2.6(1.3–6.7)	2.0(1.3–3.6)	2.7(0.8–6.8)	2.0(1.1–3.3)	2.6(1.0–6.9)
Age at first intercourse	**-**	**-**	**-**	**-**	1.8(1.2–3.9)	2.7(0.9–6.9)	2.1(1.4–3.4)	2.7(1.1–6.3)
Life time sexual partner	**-**	**-**	**-**	**-**	**-**	**-**	2.1(1.4–3.5)	2.6(1.1–6.4)
**CD4 cell count <200 cells/mm^3^** *	3.2(1.5–6.6	4.0(1.2–12.6)	3.5(2.1–7.5)	4.1(3.5–11.6)	3.7(2.0–7.1)	4.6(3.2–10.1)	2.0(1.0–6.1)	4.9(3.0–12.1)
Age at first intercourse	**-**	**-**	3.3(2.0–6.5)	4.2(2.8–10.9)	3.1(1.9–9.3)	4.7(0.9–11.1)	2.7.1(1.7–9.1)	5.1(3.4–10.1)
Life time sexual partner	**-**	**-**	**-**	**-**	3.0(0.9–10.1)	4.2(0.9–9.4)	2.8.1(0.9–9.2)	5.0(3.1–11.3)
Antiretroviral drug use^e^	**-**	**-**	**-**	**-**	**-**	**-**	1.9(1.1–5.9)	5.7(1.1–7.2)
**HIV viral load above 1000 copies/ml** *	2.0(1.2–3.4)	2.8(1.1–6.8)	2.0(1.0–3.2)	2.8(1.1–6.6)	1.8(1.1–3.1)	2.8(1.2–6.9)	1.9(1.1–3.6)	2.8(1.1–4.0)
Age at first intercourse			1.9(1.1–3.1)	2.8(1.1–6.8)	1.9(1.0–3.4)	2.7(1.0–6.6)	1.8(0.9–3.4)	2.6(1.0–4.5)
Life time sexual partner					1.9(1.0–3.5)	2.7(1.2–6.4)	1.9(1.1–3.6)	2.7(1.0–5.3)
Antiretroviral drug use							1.9(0.8–3.7)	2.7(0.7–5.7)

a Age groups: 18–35 and ≥35 (ref) years;

b Marital status: Married and unmarried (ref);

c Age at first intercourse: <20 (ref) and ≥20 years;

d Life time sexual partner: 1 (ref) and ≥2;

e Antiretroviral drug use: Yes(ref) and No.

*HIV positive women only.

HIV positive women with CD4 cell count <200 cells/mm^3^ were also found to be at increased risk for SIL (aOR: 1.9; 95% CI: 1.1–5.9) and HSIL (aOR: 5.7; 95% CI: 1.1–7.2) respectively, compared to women with CD4 cell count above 200 cells/mm^3^ after adjustment for confounders in the three models. HIV positive women who were not on ARV treatment were found to be at increased risk for both SIL (aOR: 2.1; 95% CI: 1.4–3.5) and HSIL (aOR: 2.6; 95% CI: 1.1–6.4), compared to women on HAART. HIV viral load above 1000 copies did not retain its independent association with SIL (aOR: 1.9; 95% CI: 0.8–3.7) or HSIL (aOR: 2.7; 95% CI: 0.7–5.7) after adjustment was made for the use of antiretroviral drugs in the final model (table 3).

## Discussion

The prevalence of cervical squamous intraepithelial lesion (SIL) in this study was 8.4%, with a higher prevalence of 14.3% in HIV positive women compared to 3.3% in HIV negative women (aOR: 5.4; 95% CI: 2.9–8.8). HIV positive women not on antiretroviral drugs were found to be at a higher risk for both SIL (aOR: 2.1; 95% CI: 1.4–3.5) and HSIL (aOR: 2.6; 95% CI: 1.1–6.4) compared to those on antiretroviral drugs. Severely immune-compromised women (CD4 <200 cells/mm^3^) were also found to be at a higher risk for the development of both SIL (aOR: 1.9; 95% CI: 1.1–5.9) and HSIL (aOR: 5.7; 95% CI: 1.1–7.2) compared to those with CD4 cell count above 200 cells/mm^3^. No association was found between HIV viral load and SIL (aOR: 1.9;95% CI: 0.8–3.7).

Anorlu et al [23], Durowede et al [24] and Pimentel et al [25] in their separate studies reported lower SIL prevalence of 5.0%, 5.0% and 6.8%, respectively, in Lagos, Okene and Olufadi in Nigeria. The difference between the findings of these studies and our study may be due to the large proportion of HIV positive persons in our cohort. In the presence of HIV infection and the associated immune-depression, the body is unable to eliminate HPV leading to the persistence of oncogenic HPV genotypes, which ultimately results in the development of precancerous lesions and invasive carcinoma [27]. The finding of significantly higher rate of SIL among the HIV positive women (14.3%) compared to the HIV negative (3.3%) in this study further supports the previously reported association between HIV, HPV and cervical precancerous lesions [15], [16], [28]–[30]. The prevalence of SIL among HIV positive women in this study is within the range of 10.9–17.8% reported among HIV positive women by Anorlu et al [31], Sewande et al [22] and Chalermchockcharoenkit et al [32] in Nigeria and Thailand but lower than rates reported among the HIV-positive women in Jos, Nigeria (29%) [19] and Helsinki Finland (33%) [33].

The risk of developing SIL and HSIL was found to be higher in HIV positive women with severe immunosuppression, which is in support of previous studies from southern Africa, North America and Asia that reported higher prevalence of SIL among severely immune-compromised women [13]–[16]. The independent association between SIL and severe immunosuppression was retained after controlling for the use of antiretroviral drugs, confirming the independent effect of severe immunosuppression with poor clearance of HPV from cervical cells and eventual transformation of SIL to invasive cancer [14], [17], [18], Agaba et al [19] and Sewande et al [22] reported that the immunosuppressive effect, measured by the decrease in CD4 cell count, is the greatest predictor of cervical lesion development in HIV-positive women. Unlike the observed association between low CD4 count and SIL, the association between high HIV viral load and risk of SIL was not retained after adjustment was made for use of antiretroviral drugs. This suggests that HIV viral load as a factor does not influence the development of SIL, rather the initially observed association may be due to high viral load among participants not on HAART. Confirming further the protective effect of antiretroviral drugs against the development of SIL [14], [17], [18], data from this study showed an increased risk of both SIL (aOR: 2.1; 95% CI: 1.4–3.5) and HSIL (aOR: 2.6; 95% CI: 1.1–6.4) among participants not on HAART. Similar observation was reported by Firnhaber and colleagues in South Africa [12]. Antiretroviral drugs, by controlling HIV replication and reversing the weakening of the immune response and system, reduces the risks of development of cervical lesion and their progression to invasive cancer by enhancing the cervical cells ability to eliminate HPV infection and prevent persistence [27].

Our study is the first in Nigeria and the West African sub region to show a beneficial effect of antiretroviral drug use in reducing the risk of cervical squamous intraepithelial lesion. This finding could be important, in that the West African sub region has a specific mix of HIV types and strains and findings from elsewhere may not easily be extrapolated. Secondly studies have shown that HIV type and strains influence disease progression, interactions and outcome [34].

In low income countries, including Nigeria, where integration of cervical cancer screening into HIV care has been a major challenge [35], the possible protective effect of antiretroviral drugs against SIL found in this study could be used as advocacy tool for service integration. This finding could also add impetus to the current global public health strategy of test and treat in HIV care as a means of prevention of HIV transmission. Its implementation will in addition to reducing new HIV infection, also reduce cervical cancer incidence among HIV positive women.

Though our study has contributed new information to the body of knowledge on the relationship between HIV infection, immunosuppression, antiretroviral drug use and cervical squamous intraepithelial lesion, it needs to be interpreted with caution as it was a cross-sectional study conducted in one of the six regions of Nigeria. However, the cosmopolitan nature of the south western region, the sociocultural and ethnic diversity similar to what is obtained nationally might infer a fair representation of the situation in the whole country. The obtained information might therefore be reasonably generalizable. The long recruitment period for this study may have addressed the lack of diversity and variation associated with cross-sectional studies. The recruitment of most of HIV positive women from NIMR and the majority of HIV negative women from the community may have introduced some selection bias, however over 65% of these HIV positive women come from the same community as the HIV negatives. In addition, this strategy was considered feasible to enrol an adequate number of HIV positive participants due to the low HIV prevalence in Lagos and Ogun states.

The study has various strengths, of which diversity in the type of women recruited in the study is an important one. The inclusion of women of diverse characteristics including HIV positive and negative women, rural as well as urban women, treatment naïve and experienced women, severely immune-compromised and immunocompetent HIV positive women, could make the generalisation of the findings easier. The large number of HIV positive women in this study made it possible to conclusively demonstrate the effect of the stage of HIV infection and treatment on the prevalence and burden of SIL.

The use of midwives in the implementation of the study related activities was found to be a useful strategy. We noted that with support, supervision and institution of quality assurance system, tasks originally belonging to the medical profession may successfully be shifted to another cadre in order to meet the demand-response in resource poor public health settings [35], [36]. Improving access to antiretroviral drugs among HIV positive women as cervical cancer prevention in remote regions of our sub region requires shifting this task to the nurses and midwives in the primary and secondary health facilities.

## Conclusion

HIV infection and severe immunosuppression were found to be associated with increased risk of cervical squamous intraepithelial lesion but high viral load was not. For the first time, in West African sub-region with specific HIV type and strains, we established that antiretroviral drug use protects against the development of SIL.

Integration of cervical cancer screening programme into HIV services and early initiation of antiretroviral drug in HIV positive women especially those with severe immune-suppression could therefore prove to be useful in preventing and controlling cervical cancer development in HIV positive women.
